# Extracellular signal-regulated kinase signaling-mediated induction and interaction of FOXO3a and p53 contribute to the inhibition of nasopharyngeal carcinoma cell growth by curcumin

**DOI:** 10.3892/ijo.2014.2420

**Published:** 2014-05-06

**Authors:** JINGJING WU, QIN TANG, SHUNYU ZHAO, FANG ZHENG, YAN WU, GE TANG, SWEI SUNNY HAHN

**Affiliations:** Laboratory of Tumor Molecular Biology and Targeted Therapies of Chinese Medicine, University of Guangzhou Traditional Chinese Medicine, Guangdong Provincial Hospital of Chinese Medicine, Guangzhou 510120, P.R. China

**Keywords:** curcumin, nasopharyngeal carcinoma cells, ERK1/2, FOXO3a, p53

## Abstract

Curcumin, one of the main bioactive components extracted from a traditional Chinese medicinal herb, exhibits potent anticancer activity against many types of cancer cells including nasopharyngeal carcinoma (NPC). However, the detailed molecular mechanism underlying this is not clearly understood. In this study, we showed that curcumin significantly inhibited the growth of NPC cells in a dose-and time-dependent manner as determined by MTT assays, while increasing apoptosis was also observed as measured by flow cytometry for the FITC-Annexin V and propidium iodide (PI) label and Hoechst 33258 staining. To further explore the potential mechanism, we showed that curcumin increased the phosphorylation of ERK1/2 but not p38 MAPK in a time-dependent manner, and induced protein expression of the tumor suppressors FOXO3a and p53 in a dose-dependent manner, which were not observed in the presence of PD98059, an inhibitor of ERK1/2. Furthermore, silencing of FOXO3a and p53 genes by siRNAs overcame the inhibitory effect of curcumin on cell proliferation. Silencing or blockade of p53 using siRNA or chemical inhibitor abrogated the effect of curcumin on expression of FOXO3a protein; silencing or overexpression of FOXO3a had no further effect on curcumin-induced p53 protein expression. Furthermore, blockade of ERK1/2 and exogenous expression of FOXO3a restored the effect of curcumin on growth of cells. Together, our studies show that curcumin inhibits growth and induces apoptosis of NPC cells through ERK1/2-mediated increase in the protein expression and interaction of p53 and FOXO3a. p53 is upstream of FOXO3a, which form a regulatory loop that mediates the effect of curcumin. This study unveils a new mechanism by which curcumin inhibits the proliferation and induces apoptosis of human NPC cells.

## Introduction

Nasopharyngeal carcinoma (NPC) is one of the most common cancers of the head and neck, particularly in southern China and Southeast Asia (1,2). Though NPC patients are sensitive to radio/chemo-therapy, treatment failure remains high due to the development of local recurrence and distant metastasis (1). Epstein-Barr virus (EBV) infection, environmental factors and genetic susceptibility are associated with this malignancy (2). To date, the molecular mechanisms related to the progression and clinical outcome of NPC have not yet been fully understood. A combination of radiotherapy and adjuvant chemotherapy has been successful for certain NPC patients, but the 5-year survival rate is only 50–60%. There is almost no effective treatment for those who are resistant to radiotherapy and have tumor recurrence. Therefore, we need to further explore the pro-oncogenic pathways that lead to or promote NPC with the intention of identifying new targets for therapy and improving patient survival. In addition, understanding the molecular mechanisms and key regulatory factors exploitable in developing adjuvant therapies to augment currently available treatment protocols that allow decreased side-effects and toxicity without compromising therapeutic efficacy are also required. Recently, much attention has been focused on the use of traditional Chinese medicinal herbs in cancer prevention and therapy due to the pleiotropic effects of these agents on multiple carcinogen-activated oncogenic pathways, and their equally excellent safety profiles. Curcumin is one such potential candidate.

Curcumin, one of the main bioactive components extracted from a traditional Chinese medicinal herb, is associated with a variety of functions such as immunomodulatory, anti-inflammatory and anticancer activities (3). Substantial evidence has demonstrated that curcumin acts as a potential chemopreventive agent as well as a novel adjuvant treatment agent for head and neck malignancies via its effect on a variety of biological pathways (4). However, the detailed mechanisms by which this agent is involved in the inhibition of NPC cell growth and induction of apoptosis have not been well elucidated.

The p53 tumor suppressor is a major regulator of cell proliferation and apoptosis, which is activated in response to DNA damage and is mutated in ∼50% of human cancers (5). As a tumor suppressor protein, p53 plays a pivotal role in regulating the cellular response to stress and damage signals, and loss of p53 functionality is common in more than 50% of cancers (6). The role of p53 in the link of NPC has been reported, activation of p53 was involved in the radioresponse in NPC (7) and played an important role in the development of novel therapies for NPC treatment (8). The forkhead box, class O belongs to the family of mammalian forkhead transcription factors, including FOXO3a (or FKHRL1), FOXO1a (or FKHR), and FOXO4a (or AFX), which are regulated by growth factor receptor-induced activation of the phosphatidylinositol 3-kinase (PI3K)/AKT (or protein kinase B) signaling pathway (9). Studies in mammalian cells have shown that activation of FOXO3a by stimulated the expression of proteins that are involved in apoptosis (9) and cell cycle arrest (10) in different types of cells. FoxO3a is a transcription factor with known tumor suppressor activity and a conserved 110-amino acid DNA-binding domain and recognize two consensus DNA-binding sequences: 5′-TTGTTTAC-3′ and 5′-(C/A)(A/C)AAA(C/T)AA-3′ (11). Inhibition of FOXO3a expression promotes cell transformation, tumor progression and angiogenesis (12). Expression of FOXO3a was reported in NPC and considered as important prognostic marker in NPC (13).

In the present study, we investigated the potential mechanism by which curcumin inhibited NPC cell growth and induced apoptosis. Our results showed that extracellular signal-regulated kinase 1/2 (ERK1/2) signaling pathway-mediated increase in the protein expression and the interaction of FOXO3a and p53 contributed to the effect of curcumin on proliferation and apoptosis of NPC cells.

## Materials and methods

### Reagents

Curcumin was purchased from Sigma-Aldrich (Shanghai, China). RPMI-1640 and penicillin/streptomycin were purchased from Invitrogen (Carlsbad, CA, USA), fetal bovine serum (FBS) was purchased from HyClone (UT, USA). Monoclonal antibodies specific for p38 mitogen-activated protein kinase (p38 MAPK); ERK1/2 and their phosphor-forms, and FOXO3a and p53 were purchased from Cell Signaling Technology (Beverly, MA, USA). The GAPDH, p53 and FOXO3a monoclonal antibodies were obtained from Abcam Co (Burlingame, CA, USA). PVDF membrane was purchased from Millipore (USA). PD98059 (a special inhibitor of ERK1/2) was purchased from Merck Millipore (Darmstadt, Germany), MTT powder and pifithrin-α (a special inhibitor of p53) were purchased from Sigma-Aldrich (St. Louis, MO, USA). p53 and FOXO3a siRNAs were obtained from Santa Cruz (CA, USA). Lipofectamine 2000 reagent was purchased from Invitrogen (Shanghai, China). The FOXO3a-GFP and N1-GFP plasmids were kindly provided by Frank M.J. Jacobs (Rudolf Magnus Institute of Neuroscience, Department of Pharmacology and Anatomy, University Medical Center, Utrecht) and was reported previously (14). Annexin V-FITC Apoptosis Kit was purchased from Bestbio Co. (Shanghai, China).

### Cell culture

Human NPC cell lines CNE2 and HNE2 were obtained from the Cell Line Bank at the Laboratory Animal Center of Sun Yat-sen University starting March, 2012 (Guangzhou, China). All cell lines have been tested and authenticated for absence of mycoplasma, genotypes, drug response, and morphology using a commercially available kit (Invitrogen, Shanghai, China) in the Laboratory Animal Center at Sun Yat-sen University in April, 2010. Cells were cultured in RPMI-1640 medium, supplemented with 10% fetal bovine serum (FBS), 5% glutamine, 100 U/ml penicillin and 100 mg/ml streptomycin. In all experiments, 60–70% of confluent cells were washed and incubated with curcumin, PD98059, pifithrin-α for the indicated time. Curcumin was dissolved in DMSO and the final concentration was <1‰ (v/v) in all experiments.

### MTT assay

Cell viability was analyzed by the MTT [3-(4, 5-dimethylthiazol-2-yl)-2, 5-diphenyl tetrazolium bromide] assay. Briefly, cells were seeded in 96-well plates at the density of 2×10^3^ cells/well and were cultured with increased concentrations of curcumin for up to 72 h, and then 10 *μ*l of 10 mg/ml MTT solution was added to each well for an additional 4 h according to the manufacturer’s instructions (Promega, Shanghai, China). After centrifugation, 150 *μ*l dimethyl sulfoxide was added to the precipitate and the absorbance of the enzyme was measured at 570 nm using a Microplate Reader (Bio-Rad, Hercules, CA, USA). Cell growth rates (average absorbance of each treat group and non-treated group) were then calculated. All experiments were performed in triplicate and repeated at least three times.

### Hoechst 33258 staining

Following treatment with curcumin at various concentrations for up to 48 h, cells were washed twice with PBS and fixed in 1 ml of 4% paraformaldehyde for 10 min at 4°C. After washing twice with PBS, cells were stained with 100 *μ*l (10 *μ*g/ml) Hoechst 33258 (Sigma, St. Louis, MO, USA) in PBS for 15 min at room temperature in the dark, and then washed with PBS. Afterwards, the cells were mounted and examined under fluorescence microscopy (Olympus IX71, Tokyo, Japan). Apoptotic cells were identified by the condensation and fragmentation of their nuclei.

### Flow cytometry

Cells (1×10^5^ cells/dish) were seeded in 6-mm culture plate and treated with increased doses of curcumin as indicated for up to 48 h. The adherent and floating cells were both collected and resuspended in cold PBS for analysis. Cells were stained with Annexin V-FITC apoptosis detection kit to monitor apoptosis cells and propidium iodide (PI) to detect dead cells according to the instructions from the provider. Samples were analyzed on the FC500 Flow cytometry Systems (Beckman Coulter, USA).

### Western blot analysis

After being treated with curcumin, cells were harvested and washed with ice-cold phosphate buffer, homogenized in 1X RIPA lysis buffer. Equal amounts of protein from whole cell lysates were solubilized in 2X SDS-sample buffer, separated on SDS-polyacrylamide gels. The separated proteins were transferred onto nitrocellulose using a Bio-Rad Trans Blot semidry transfer apparatus for 1 h at 25 V, blocked with Blotto with 5% no-nfat dry milk and 0.1% Tween-20 overnight at 4°C, and washed with wash buffer. Blots were incubated with polyclonal antibodies against p38 MAPK, ERK1/2 and their phosphor-forms, and FOXO3a and p53 (1:1,000) overnight at 37°C, washed and incubated with a secondary antibody raised against rabbit IgG conjugated to horseradish peroxidase (1:2,000, Sigma, Beijing, China) for 1 h at room temperature. The washed blots were transferred to freshly made ECL Prime (Pierce, Rockford, IL, USA) and exposed to X-ray film.

### Treatment with p53 and FOXO3a small interfering RNAs

For the transfection procedure, cells were grown to 60% confluence, and p53 and FOXO3a siRNAs and control siRNA were transfected using the oligofectamine reagent (Invitrogen). CNE2 cells (2×10^5^ cells/ml) were seeded in RPMI-1640 medium without antibiotics overnight. After washing the cells with PBS, 2 ml of media without antibiotics were added. Thereafter, 200 *μ*l of Lipofectamine 2000 complex was added into each plate. The cells were transfected with control or FoxO3a (FKHRL1) and p53 small interfering RNAs (siRNAs) for 24 h according to instructions from the manufacturer. At 24 h after transfection, medium was replaced for complete DMEM and cells were transfected with either exogenous *FOXO3a* gene using electroporated transfection method or treated with curcumin 40 *μ*M for an additional 24 h for other experiments.

### Electroporated transfection assays

CNE2 cells (1×10^7^ cells/ml) were transferred into conical tubes and centrifuged at 1,200 rpm for 5 min. After centrifuging, the medium was removed and the cells were washed with 1X PBS, and centrifuged again at 1,200 rpm for 5 min. Afterwards, the PBS was aspirated and added to Bio-Rad Gene Pulser electroporation buffer. After resuspending the cells, the desired N1-GFP or FoxO3a-GFP plasmid DNA at a final concentration of 10 to 20 *μ*g/ml was added and the electroporation plate was put in the MXcell plate chamber. The electroporation conditions on the plates to deliver 160 V/10 ms square wave were adjusted until reaching the optimum. The conditions were set and loaded onto the device Gene Pulser II Electroporation System (Bio-Rad, CA, USA). After electroporation was completed, the cells were transferred to a tissue culture plate. We typically transferred each 150 *μ*l electroporation sample to a 6-well tissue culture plate containing 2 ml RPMI-1640. Cells were then incubated for 48 h at 37°C, then treated with curcumin for an additional 24 h.

### Statistical analysis

Analysis of variance and Student’s t-test were used to compare the values of the test and control samples. P<0.05 was considered a statistically significant difference. SPSS software was used for all statistical analysis. The significance was evaluated by the paired t-test. All the experiments were performed at least three times, and mean values and standard deviation were calculated.

## Results

### Curcumin inhibits proliferation of NPC cells in a dose- and time-dependent manner

**Figure 1.**
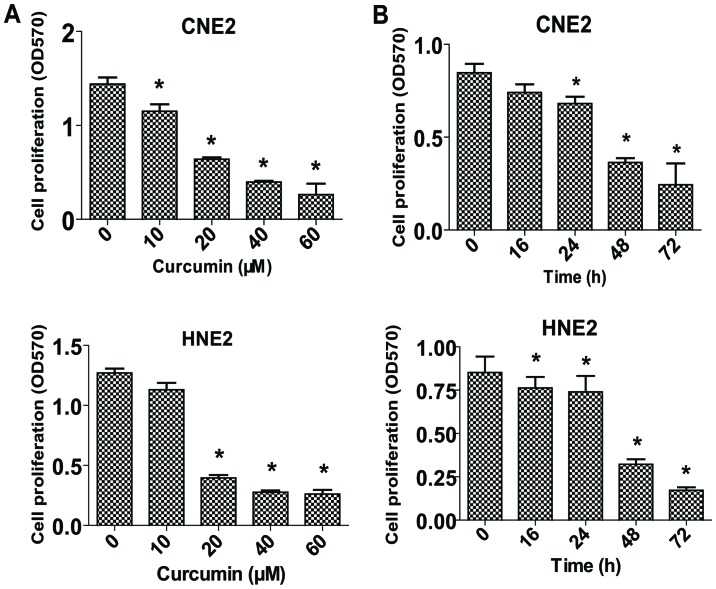
Curcumin inhibits proliferation of NPC cells. (A) Human CNE2 and HNE2 NPC cells were treated with increased concentrations of curcumin and indicated for 48 h (A) or at the concentration of 40 *μ*M for up to 72 h (B). Afterwards, cell proliferation was determined by MTT assays. The data are presented as mean ± SD of three separate experiments. ^*^P<0.05 indicates significant difference as compared to the untreated control group.

To determine whether curcumin may regulate the proliferation of NPC cells, we examined the effect of curcumin on cell proliferation in two human NPC cell lines, CNE2 and HNE2, by a MTT assay. The results showed that treatment with increased concentrations of curcumin for up to 72 h significantly inhibited cell proliferation in a dose- and time-dependent manner (Fig. 1A and B).

### Curcumin increases apoptosis of CNE2 cells

**Figure 2.**
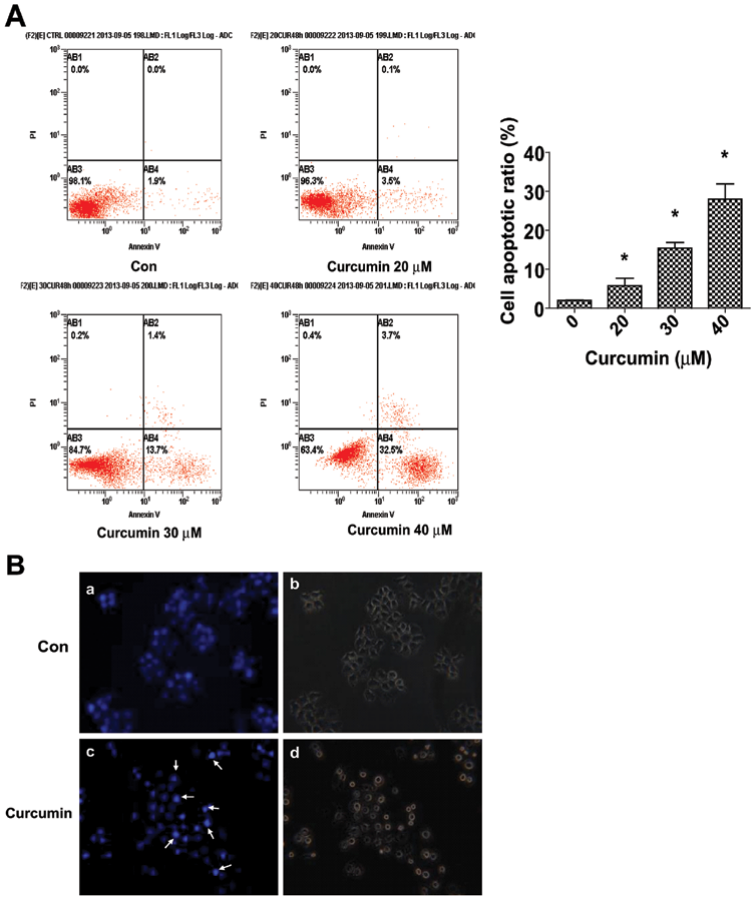
Curcumin induces apoptosis in CNE2 cells. (A) CNE2 cells were incubated with indicated doses of curcumin for 48 h, followed by staining with Annexin V/PI. The AB3 quardrant (Annexin V^−^/PI^−^), AB4 quadrant (Annexin V^+^/PI^−^) and AB2 quadrant (Annexin V^+^/PI^+^) indicate the percentage of normal cells, early apoptosis and late apoptosis, respectively. (B) Apoptotic nuclear morphology changes induced by curcumin (40 *μ*M) treatment for 48 h were observed by Hoechst 33258 staining in CNE2 cells. Panels a and c show Hoechst 33258 nuclear staining, panels b and d show cell morphology. Arrows in panel c indicate chromatin condensation and nuclear fragmentation. The data are presented as mean ± SD of three separate experiments. ^*^P<0.05 indicates significant difference as compared to the untreated control group.

We evaluated the effects of curcumin on apoptosis in CNE2 cells using Annexin V-FITC and PI staining. In normal live cells, phosphatidy serine (PS) was located on the cytoplasmic surface of the cell membrane. However, in the apoptotic cells, PS was found to be translocated from the inner to the outer leaflet of the plasma membrane, thus exposing PS to the external cellular environment. The human anticoagulant, Annexin V, is a 35–36 Kda Ca^+^-dependent phospholipid binding protein that has a high affinity for PS. Annexin V labeled with a fluorophore or biotin can identify apoptotic cells. In the scatter plot of double variable flow cytometry, AB3 quadrant (FITC^−^/PI^−^) showed living cells; AB2 quadrant (FITC^+^/PI^+^) stand for late apoptotic cells; and AB4 quadrant (FITC^+^/PI^−^) represents early apoptotic cells. As shown in Fig. 2A, a marked dose-dependent increase in both the early and late stages of apoptosis was observed in CNE2 cells after curcumin treatment compared to the control cells.

Morphological changes in the apoptotic cells were revealed by the Hoechst 33258 staining (Fig. 2B). In the untreated CNE2 cells, the nuclei were stained weakly, and homogeneously blue, whereas, in cells treated with curcumin some bright chromatin condensation and nuclear fragmentation were observed.

### Curcumin increases FOXO3a and p53 protein expression; silencing of FOXO3a and p53 abrogates the inhibitory effect of curcumin on growth of NPC cells

**Figure 3.**
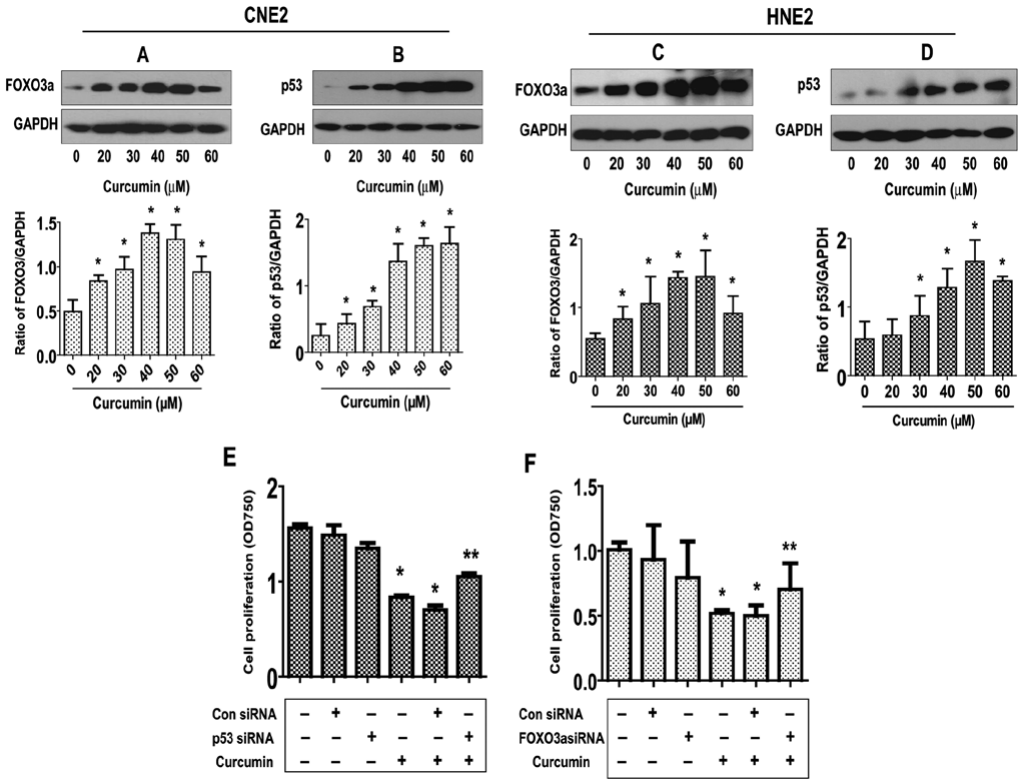
Curcumin increased FOXO3a and p53 protein expression; silencing of FOXO3a and p53 blocked the inhibitory effect of curcumin on cell growth. (A and B) Human CNE2 cells were treated with induced concentrations of curcumin as indicated for 24 h. The expression of (A) FOXO3a and (B) p53 protein were detected by western blot analysis. (C and D) Human HNE2 cells were treated with induced concentrations of curcumin as indicated for 24 h. The expression of (C) FOXO3a and (D) p53 protein were detected by western blot analysis. GAPDH was used as an internal control for loading purpose. The graphs are the densitometry results of the FOXO3a and p53 protein expression over GAPDH in at least three separate experiments. (E and F) Human CNE2 cells were transfected with control or (E) p53 or (F) FOXO3a siRNAs (100 nM each) for 24 h before exposure to curcumin (40 *μ*M) for an additional 24 h. Cell proliferation was determined by MTT assays. The data are presented as mean ± SD of three separate experiments. ^*^P<0.01 indicates significant difference from untreated control cells. ^**^P<0.05 indicates significance of combination treatment as compared with curcumin alone.

To elucidate the potential molecular mechanism underlying this effect, we tested the effect of curcumin on the expression of p53 and FOXO3a active proteins, these tumor suppressors have been shown to be involved in cell proliferation and apoptosis (5,12). As shown in Fig. 3A and B, curcumin induced the protein expression of both FOXO3a (Fig. 3A) and p53 (Fig. 3B) dose-dependently in CNE2 cells, with maximal induction observed at 40–50 *μ*M, respectively. Note that similar results were also observed in an additional cell line (HNE2) (Fig. 3C and D).

To further explore the role of these tumor suppressors in mediating the curcumin-inhibited NPC cell proliferation, we blocked p53 and FOXO3a gene expression by transfecting CNE2 cells with p53 and FOXO3a siRNAs and evaluated the effects of curcumin on cell proliferation. We demonstrated that the knockdown of p53 and FOXO3a genes significantly overcame the curcumin-mediated growth inhibition of CNE2 cells (Fig. 3E). This indicated important roles of p53 and FOXO3a expression in curcumin-inhibited cell growth.

### Curcumin induces ERK/MAPK activation, blockade of ERK1/2 abrogated the effect of curcumin on FOXO3a and p53 expression, while restoring the cell growth in the presence of curcumin in NPC cells

**Figure 4.**
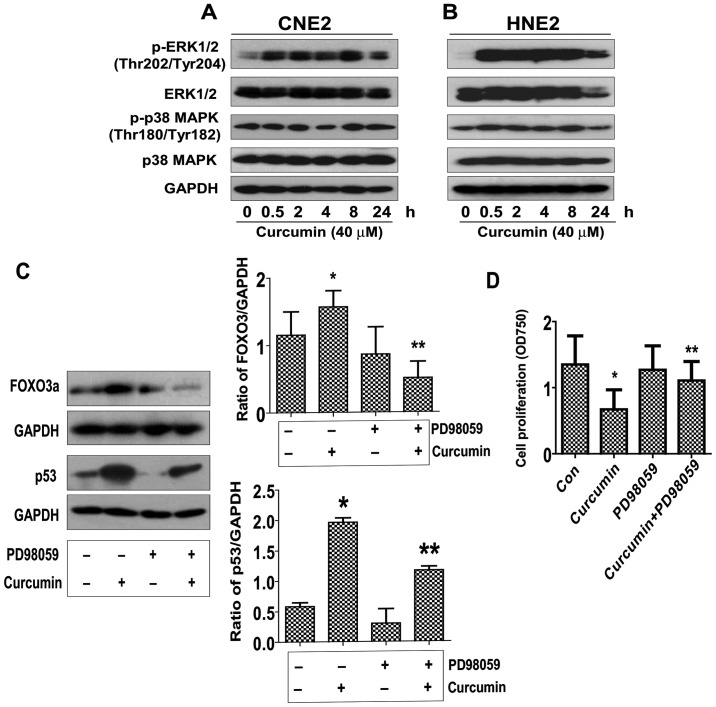
Curcumin induces ERK/MAPK activation, blockade of ERK1/2 abrogates the effect of curcumin on FOXO3a and p53 expression, while restoring the cell growth in the presence of curcumin in NPC cells. (A and B) Human CNE2 and HNE2 cells were treated with curcumin at the concentration of 40 *μ*M for up to 24 h. The expression of the phosphorylated or total protein of ERK1/2, p38 MAPK were measured by western blot analysis using corresponding antibodies. GAPDH was used as loading control. (C) Human CNE2 cells were treated with PD98059 for 2 h, followed by exposure to curcumin (40 *μ*M) for an additional 24 h. The expression of p53 and FOXO3a proteins were detected by western blot analysis using specific antibodies. The bar graphs represent the densitometry results of p53 and FOXO3a/GAPDH as mean ± SD of at least three separate experiments. (D) Human CNE2 cells were treated with PD98059 for 2 h, followed by exposure to curcumin (40 *μ*M) for an additional 24 h. Cell proliferation was determined by MTT assays. The data are presented as mean ± SD of three separate experiments. ^*^P<0.01 indicates significant difference from untreated control cells. ^**^P<0.01 indicates significance of combination treatment as compared with curcumin alone.

In order to determine the potential signaling pathways that are involved in the curcumin-mediated regulation of FOXO3a and p53 expression, thereby controlling cell proliferation, we initially evaluated the effect of curcumin on the activation of ERK and p38 MAPK by western blot analysis. As shown in Fig. 4A, treatment with curcumin at 40 *μ*M significantly increased the phosphorylation of ERK1/2 starting at 30 min and continued for up to 24 h, whereas the level of total ERK1/2 protein was unchanged. However, curcumin had little effect on the activation and protein expression of p38 MAPK. Similar results were also observed in an additional NPC cell line (HNE2) (Fig. 4B). Interestingly, we showed that the ERK inhibitor (PD98059) exposed to the cells abolished the curcumin-induced FOXO3a and p53 protein expression in CNE2 cells (Fig. 4C). Moreover, treatment of PD98059 restored the cell growth in the presence of curcumin (Fig. D). Together, the above results suggested that activation of ERK signaling pathway was critical in mediating the effect of curcumin on regulating FOXO3a and p53 expression, and cell growth.

### p53 is upstream of FOXO3a; exogenous expression of FOXO3a restores the effect of curcumin on growth of cells silenced by endogenous FOXO3a gene

**Figure 5.**
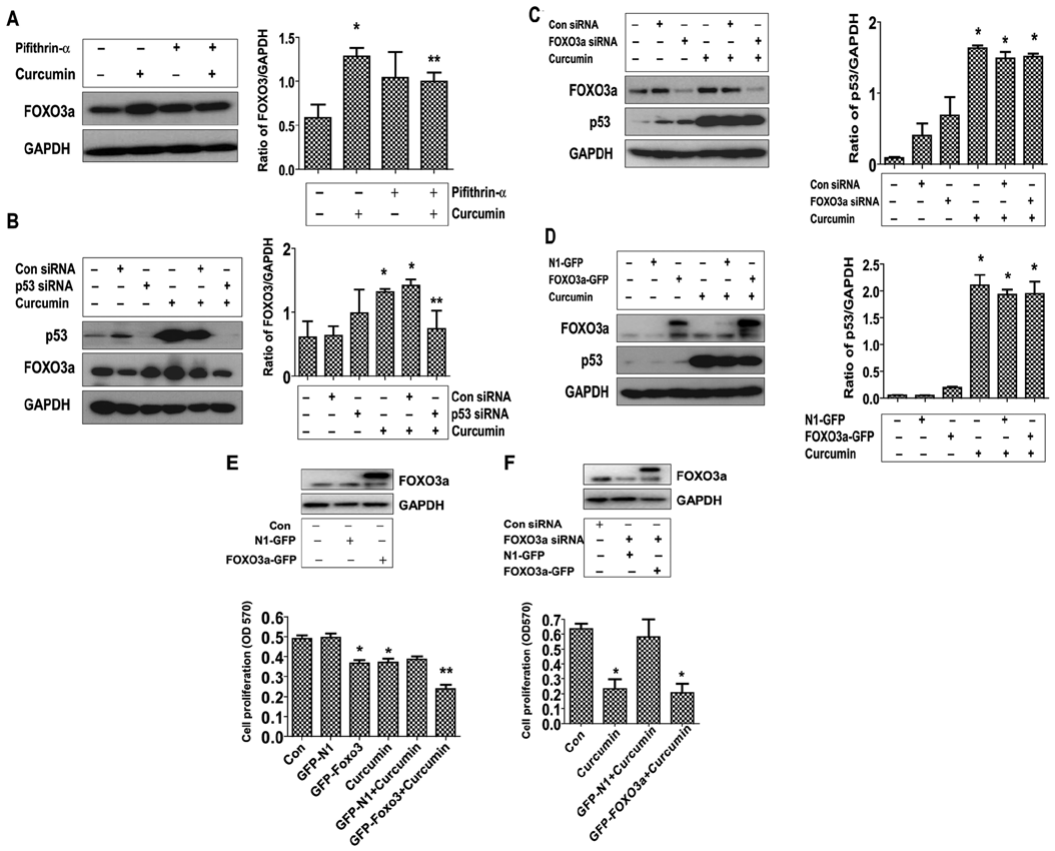
p53 is upstream of FOXO3a. Exogenous expression of FOXO3a restored the effect of curcumin on growth of cell silenced by endogenous *FOXO3a* gene. (A) CNE2 cells were treated with p53 inhibitor Pifithrin-α (10 *μ*M) for 2 h, followed by exposure to curcumin (40 *μ*M) for an additional 24 h. The expression of p53 and FOXO3a was detected by western blot analysis. (B) CNE2 cells were transfected with control or p53 siRNAs (100 nM) for 24 h, followed by exposure to curcumin (40 *μ*M) for an additional 24 h. The expression of p53 and FOXO3a protein was detected by western blot analysis. (C) CNE2 cells were transfected with control or FOXO3a siRNAs (100 nM each) for 24 h before exposure to curcumin (40 *μ*M) for an additional 24 h. The expression of p53 was detected by western blot analysis. (D) CNE2 cells were transfected with control (pEGFP-N1) or FOXO3a expression vector (FOXO3a-pEGFP) for 24 h, and then treated with curcumin (40 *μ*M) for an additional 24 h. The expression of p53 and FOXO3a protein was detected by western blot analysis. (E) CNE2 cells were transfected with control (pEGFP-N1) or FOXO3a expression vector (FOXO3a-pEGFP) for 24 h before exposure to curcumin (40 *μ*M) for an additional 24 h. (F) CNE2 cells were transfected with control or FOXO3a siRNA for 30 h, followed by control (pEGFP-N1) or FOXO3a expression vector (FOXO3a-pEGFP) for up to 24 h before exposure to curcumin (40 *μ*M) for an additional 24 h. Cell proliferation was determined by MTT assays. The upper insert panels are blots of expression of FOXO3a protein detected by western blot analysis. ^*^P<0.01 indicates significant difference from untreated control cells. ^**^P<0.05 indicates significance of combination treatment as compared with curcumin alone.

Transcriptional activity of FOXO3a could be modulated though interactions with other transcriptional factors such as tumor suppressor p53 (15), thus, we assessed if curcumin affected the interactions of FOXO3a with p53. As shown in Fig. 5, silencing and blockade of p53 using either siRNA or specific p53 inhibitor overcame the induced effect of curcumin on the expression of FOXO3a protein (Fig. 5A and B). On the contrary, silencing or overexpression of FOXO3a had no effect on curcumin-induced p53 protein expression (Fig. 5C and D), but strengthened the inhibitory effect of curcumin on cell growth (Fig. 5E). Interestingly, exogenous expression of FOXO3a restored the effect of curcumin on growth of cells silenced by endogenous FOXO3a gene (Fig. 5F). Note that western blot analysis confirmed the exogenous expression of FOXO3a protein transfected into the cells (Fig. 5D–F).

## Discussion

Studies have shown that curcumin can inhibit the growth of a variety of tumor cells, as well as induce cell apoptosis in several tumors including NPC cells (16–20) suggesting that curcumin can be used as a natural antitumor agent. However, the detailed mechanisms by which this agent targets NPC cancer cells remained unclear. In this study, we evaluated the response of NPC cells to curcumin treatment. Our results indicated that curcumin inhibited NPC cell proliferation and induced apoptosis in a dose- and time-dependent manner suggesting a tumor suppressor property of this agent. Of note, the concentrations of curcumin used here were consistent with or even lower then those reported by others demonstrating significant responses in different cell systems (21–23) although lower doses were also reported in other studies (24,25). We realized that a higher dose was needed to inhibit different cancer cell growth, but this was within the range of those reported by others and showed no toxicity (21–23).

In our study we demonstrated the role of p53 and FOXO3a protein induction that mediated the effect of curcumin on the inhibition of NPC cell growth. As tumor suppressors, both transcription factors play important roles in several areas including gene regulation, cell growth and apoptosis (5,12). Striking similarities have been reported between p53 and FOXO, such as post-translational modifications, common signaling pathways, target genes, and similar mutual interactions with various proteins (26). We found that blockade of the activity of p53 or silencing of either p53 or FOXO3a gene partially overcame the inhibitory effect of curcumin on NPC cell proliferation, suggesting that induction of these two molecules contributed to mediation of the effect of curcumin on NPC cell growth inhibition. Whether curcumin affected the post-translational modifications, such as phosphorylation, acetylation and ubiquitination, of either p53 or FOXO3a, thereby regulating their subcellular localization and transcriptional activities require further study. Consistent with this, other studies also found the link of curcumin and p53 or FOXO3a expression, and demonstrated the role of these transcription factors in mediating the effect of curcumin on controlling cell proliferation and other functions in other cell systems (27,28). We reasoned that more studies are required to explore the precise mechanism of p53 and FOXO3a expression, regulation and downstream pathways in mediating the overall response of curcumin.

The MAPK signaling pathway plays a key role in the regulation of gene expression, cellular growth and survival (29). Data from others indicated that curcumin activates MAPK signaling pathways, and that activation of MAPK, such as ERK and p38 MAPK, links curcumin-mediated signaling to the transcriptional regulation of genes that are crucial for cell growth inhibition (30,31). Our result identified an important role of ERK activation in mediating the inhibitory effect of curcumin on p53 and FOXO3a protein expression and NPC cell growth inhibition. However, p38 MAPK played no role in our study, which differed from others (31,32). The discrepancy remain unclear, the use of different cell lines and the culture conditions may account for this, which needs to be further determined. We reasoned that curcumin may function through activating ERK/MAPK signaling pathway, followed by upregulation of p53 and FOXO3a protein expression, thereby inhibiting NPC cell proliferation and inducing apoptosis. However, inhibition of ERK/MAPK reversed the effect of curcumin on NPC proliferation and could be independent of p53 and FOXO3a signaling. Also, the potential downstream targets of p53 and FOXO3a may be involved in this process. Thus, more studies are required to further elucidate this.

The aforementioned uncovered that both p53 and FOXO3a played a causative role in mediating the effect of curcumin in controlling NPC cell growth and perhaps inducing apoptosis. Furthermore, our results showed that exogenous expression of FOXO3a could enhance the effect of curcumin on cell growth, and more importantly, restore the inhibitory effect of curcumin on growth of NPC cells silenced by endogenous FOXO3a gene in siRNA approach, while having no further effect on curcumin-induced p53 expression. This further confirmed the role of FOXO3a in mediating the effect of curcumin on cell growth; it also suggested that p53 is upstream of FOXO3a and induction of FOXO3a by curcumin is p53-dependent. We speculated that p53 and FOXO may have parallel functions that worked in concert to mediate the effect of curcumin on control growth of NPC cells. The p53 and FOXO3a interaction have been identified (26), however, the extent and importance of the functional interaction between these two have not been fully explored. Consistent with our report, other studies found that FOXO3a was a p53 target gene and that transcriptional activity of FOXO3a was regulated by p53, while the latter was not affected by FOXO3a (33,34). The p53 and FOXO3a formed part of regulation transcriptional network to control cancer cell growth and apoptosis (33,34). In addition, curcumin induced expression of p53 or/and FOXO3a in inhibition of cancer cell growth and other functions have been shown in other cell systems (27,28,35,36). However, whether curcumin affects the interaction of these proteins to facilitate the inhibitory effect of NPC growth remain to be determined. A number of signaling pathways mediated the expression of FOXO3a and p53, and critically regulated apoptotic events, which included PI3-K/Akt and cyclin-dependent kinase (CDK) (37,38). We believed that understanding the functional interactions of p53 and FOXO3a, their regulated signaling pathways and known downstream targets will be crucial to better elucidate their roles in mediating the overall response of curcumin.

In summary, our data show that curcumin inhibits the growth and induces apoptosis of NPC cells through activation of the ERK signaling pathway; this leads to increase in protein expression of p53 and FOXO3a. p53 is upstream of FOXO3a and induction of FOXO3a by curcumin is p53-dependent. Both transcription factors and tumor suppressors form a regulatory loop that work in concert to mediate the effect of curcumin. This study unveils a new mechanism by which curcumin inhibits the proliferation and induces apoptosis of NPC cells.
